# Maternal Oral Microbiome Dysbiosis and Adverse Pregnancy Outcomes: Microbial Signatures, Inflammatory Pathways, and Clinical Evidence

**DOI:** 10.3390/jcm15114379

**Published:** 2026-06-05

**Authors:** Eugenia-Alina Radu, Elena Mocanu, Maria Fulina, Vadym Rotar, Florin Enache, Stere Popescu, Lucian Șerbănescu

**Affiliations:** 1 County Clinical Emergency Hospital “Sf. Ap. Andrei”, 900591 Constanta, Romania; eugenia-alina.radu@rez.umfcd.ro (E.-A.R.); maria.fulina@yahoo.com (M.F.); rotarvadym.obst.gyn@gmail.com (V.R.); florin.enache@365.univ-ovidius.ro (F.E.); stere.popescu@365.univ-ovidius.ro (S.P.); lucian.serbanescu@365.univ-ovidius.ro (L.Ș.); 2 Faculty of Medicine, Ovidius University of Constanta, 900470 Constanta, Romania; 3 Santerra Medical Center, 900178 Constanta, Romania

**Keywords:** pregnancy, oral microbiome, preterm birth, low birth weight, maternal oral health, adverse pregnancy outcomes

## Abstract

**Background/Objectives**: Pregnancy is characterized by complex physiological, hormonal, and immunological changes that influence the oral environment and the microbial composition of the oral cavity. Emerging evidence suggests that maternal oral dysbiosis may be associated with systemic inflammatory responses and may potentially influence pregnancy outcomes. This systematic review aimed to evaluate the current clinical evidence regarding the association between maternal oral dysbiosis and adverse pregnancy outcomes, including preterm birth, low birth weight, and gestational complications. **Methods**: A systematic search of PubMed, Scopus, Web of Science, and Cochrane Library was conducted for studies published between January 2013 and September 2025. Observational studies and clinical trials examining the relationship between maternal oral dysbiosis or periodontal pathogens and pregnancy outcomes in pregnant women were included. Study selection was performed according to the Preferred Reporting Items for Systematic Reviews and Meta-Analyses (PRISMA 2020) guidelines, and the review was prospectively registered in PROSPERO (CRD420261383855). Data were extracted on the study design, population characteristics, microbiological assessment methods, and reported pregnancy outcomes. Ten studies met the inclusion criteria of this review. **Results**: Seven of the ten included studies reported significant associations between the increased prevalence of periodontal pathogens, including *Porphyromonas gingivalis*, *Fusobacterium nucleatum*, and *Prevotella intermedia*, and adverse pregnancy outcomes, particularly preterm birth and low birth weight (LBW). Several studies have identified oral bacterial DNA in placental tissues, supporting the potential hematogenous microbial translocation pathways. However, heterogeneity in microbiological assessment techniques and study designs limits the comparability of the findings. **Conclusions**: Current evidence suggests that maternal oral dysbiosis may be associated with the inflammatory pathways linked to adverse pregnancy outcomes. Further prospective studies and standardized microbiome analyses are required to clarify the role of the oral microbiome in maternal and fetal health. Integrating oral health assessments into prenatal care may be an important strategy for improving maternal and neonatal outcomes.

## 1. Introduction

Maternal health during pregnancy is influenced by the complex interplay of biological, behavioral, and environmental factors. Increasing attention has been directed toward the potential role of oral health in systemic conditions and pregnancy outcomes [1,2].

The oral cavity hosts a highly diverse microbial ecosystem composed of more than 700 identified bacterial species organized into complex biofilm communities that interact with host immune responses and systemic physiology [2].

For clarity, several related but distinct concepts should be differentiated from one another. The oral microbiome refers to the entire community of microorganisms inhabiting the oral cavity and their collective genetic content. Oral microbiome alterations refer to changes in the composition, diversity, or functional characteristics of this microbial community. Oral dysbiosis refers to the disruption of the normal microbial balance, typically characterized by a shift from a symbiotic state to a microbial profile associated with inflammation or disease. Periodontal pathogens are specific microorganisms, such as *Porphyromonas gingivalis* and *Fusobacterium nucleatum*, implicated in the development and progression of periodontal disease. Periodontal disease is a chronic inflammatory condition affecting the supporting structures of the teeth and is commonly associated with dysbiotic oral microbial communities.

Pregnancy is associated with significant hormonal fluctuations, particularly increases in estrogen and progesterone levels, which can influence the oral microbiome. These hormonal changes affect vascular permeability, gingival inflammatory responses, and immune regulation, creating conditions that may promote microbial shifts in the oral cavity [3]. Consequently, pregnant women frequently experience oral health conditions such as gingivitis, periodontitis, and dental caries [2,3].

Beyond local oral manifestations, growing evidence suggests that oral dysbiosis may have systemic effects. Periodontal disease, a chronic inflammatory condition associated with dysbiotic oral biofilms, has been extensively investigated for its potential relationship with adverse pregnancy outcomes [4].

Adverse pregnancy outcomes associated with maternal oral dysbiosis include preterm birth, low birth weight, and fetal growth restriction [4].

Several biological mechanisms have been proposed to explain the association between oral dysbiosis and pregnancy complications. One proposed mechanism involves the production of inflammatory mediators during periodontal infection, including prostaglandins, tumor necrosis factor-alpha, and interleukin-6. These mediators may enter the systemic circulation, influence placental tissues, and contribute to the pathways involved in premature labor [1,2]. Another proposed mechanism involves the hematogenous dissemination of periodontal pathogens or their endotoxins into the maternal bloodstream, allowing microbial colonization of the placental tissues or amniotic fluid [5,6].

Recent advances in molecular microbiology and sequencing technologies have further strengthened interest in the maternal oral microbiome. Studies have reported the presence of oral bacteria, including *Fusobacterium nucleatum*, in placental tissues and amniotic fluid samples, suggesting possible hematogenous dissemination from the oral cavity to the fetoplacental unit [7,8]. These findings support the concept that oral dysbiosis may influence systemic inflammatory processes during pregnancy.

Despite increasing research interest, the strength and consistency of the evidence linking maternal oral dysbiosis to pregnancy outcomes remains debated. Some studies have reported significant associations between periodontal pathogens and adverse outcomes, whereas others have failed to demonstrate a clear relationship [9,10]. Differences in study design, microbial detection methods, and population characteristics contribute to this variability [7,8,9].

Given the expanding literature in this area, a comprehensive synthesis of the available evidence is necessary to clarify the potential role of the maternal oral microbiome in pregnancy outcomes. Therefore, this systematic review aimed to evaluate the current clinical evidence regarding the association between maternal oral dysbiosis and adverse pregnancy outcomes.

Although previous systematic reviews have investigated the association between periodontal disease and adverse pregnancy outcomes, recent advances in oral microbiome sequencing and microbial functional analysis have considerably expanded this evidence. The present review differs from earlier reviews by specifically emphasizing oral dysbiosis, changes in microbiome composition during pregnancy, microbial translocation to placental tissues, and sequencing-based characterization of microbial communities. Furthermore, this review incorporates recent studies evaluating microbial metabolic pathways and longitudinal microbiome dynamics across pregnancy trimesters.

## 2. Materials and Methods

### 2.1. Study Design

This systematic review was conducted in accordance with the PRISMA 2020 guidelines [11] (File S1) and was prospectively registered in PROSPERO (CRD420261383855).

The objective of the review was to synthesize available evidence regarding the association between maternal oral dysbiosis and adverse pregnancy outcomes.

The research question was developed according to the Population–Exposure–Outcome (PEO) framework, where the population consisted of pregnant women, the exposure included oral microbiome dysbiosis or periodontal microbial alterations, and the outcomes included adverse pregnancy outcomes such as preterm birth and low birth weight.

### 2.2. Search Strategy

A systematic literature search was performed in the following electronic databases:

- PubMed/MEDLINE;
- Scopus;
- Web of Science;
- Cochrane Library.

The search included studies published between January 2013 and September 2025. The final database search was conducted on 15 November 2025. The search strategy was adapted for each database according to its indexing system and search interface. Complete search strategies for all databases are provided in Supplementary Table S1.

Reference lists of relevant studies and reviews were also manually screened to identify additional eligible articles.

The search included studies published between January 2013 and September 2025. The following combination of keywords and Medical Subject Headings (MeSH) terms was used: (pregnancy OR pregnant women) AND (oral microbiome OR oral microbiota OR periodontal pathogens OR oral dysbiosis OR periodontal disease) AND (preterm birth OR low birth weight OR adverse pregnancy outcomes OR preeclampsia).

The search was restricted to studies published from January 2013 onward, as this period corresponds to the broader adoption of high-throughput 16S rRNA sequencing technologies in oral microbiome research, enabling more comprehensive and reproducible microbiological characterization.

### 2.3. Eligibility Criteria

Studies were included if they met the following criteria:

1. Investigated pregnant women as the study population.
2. Evaluated oral microbiome composition, periodontal pathogens, or periodontal disease indicators.
3. Reported pregnancy outcomes, including preterm birth, low birth weight, preeclampsia, or other obstetric complications.
4. Observational studies (cohort, case–control, cross-sectional) or clinical trials.
5. Published in English.

Studies were excluded if they were:

- Animal studies;
- Case reports or case series;
- Editorials or commentaries;
- Narrative or systematic reviews;
- Studies lacking relevant pregnancy outcome data.

### 2.4. Study Selection

All records identified through the database searches were exported to a reference management software program, where duplicate records were removed. Two reviewers (E.-A.R. and L.Ș.) independently screened titles and abstracts to determine potential eligibility. Studies considered potentially relevant were retrieved for full-text evaluation.

Full-text articles were independently assessed by the same reviewers according to the predefined inclusion and exclusion criteria. Disagreements during study selection were resolved through discussion and consensus. When necessary, a third reviewer (E.M.) was consulted to resolve disagreements. Inter-reviewer agreement statistics were not prospectively recorded and therefore Cohen’s kappa could not be calculated.

A total of 1248 records were identified through database searches. After removing 314 duplicate records, 934 articles were screened based on title and abstract. Following the screening process, 112 full-text articles were assessed for eligibility. Of these, 100 studies were excluded due to insufficient microbiome data, absence of pregnancy outcome reporting, or laboratory-based study design. Finally, 10 studies were included in the qualitative synthesis.

**Figure 1:**
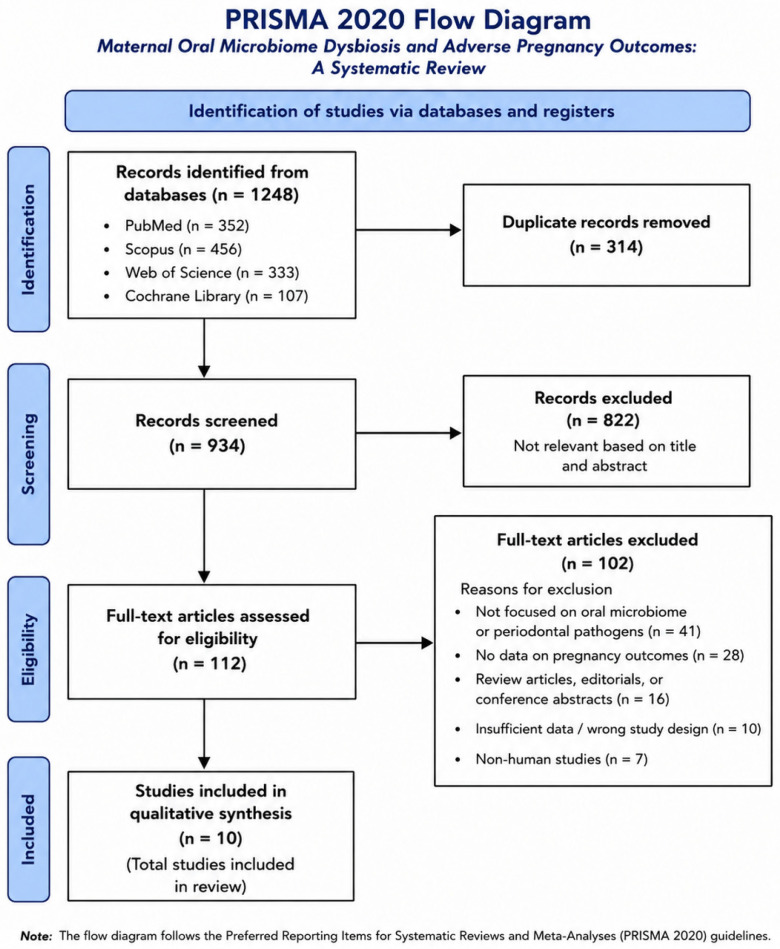
PRISMA 2020 flow diagram of the study selection process.

The study selection process is summarized in the PRISMA flow diagram (Figure 1).

### 2.5. Data Extraction

Data extraction was performed independently by two reviewers (E.-A.R. and L.Ș.) using a standardized extraction form. The extracted data were compared for consistency, and discrepancies were resolved through discussion and consensus. A third reviewer (E.M.) was consulted when required. The following information was collected:

- Author and year of publication;
- Country of study;
- Study design;
- Sample size;
- Methods used for microbiome analysis;
- Oral microbial species identified;
- Pregnancy outcomes evaluated;
- Main findings.

The extracted variables and detailed study-level data are provided in Supplementary Table S4.

Detailed study characteristics, including gestational age at sampling, sampling site, microbiological methods, and sequencing platforms, are presented in Supplementary Table S4.

### 2.6. Risk of Bias Assessment

For transparency, domain-specific ratings and the rationale for each NOS judgment are provided in Supplementary Table S3. The assessment was performed independently by two reviewers, and disagreements were resolved through discussion and consensus. This tool evaluates studies across three domains:

- Selection of study groups;
- Comparability between study groups;
- Assessment of outcomes or exposures.

Each study was assigned a score ranging from 0 to 9 points, with higher scores indicating lower risk of bias. Studies scoring 7–9 points were considered to have low risk of bias, while studies scoring 5–6 points were classified as moderate risk.

### 2.7. Certainty of Evidence Assessment

The overall certainty of evidence for the principal outcomes evaluated in this review was assessed using the Grading of Recommendations Assessment, Development and Evaluation (GRADE) framework. The certainty of evidence was categorized as high, moderate, low, or very low based on study design, risk of bias, inconsistency, indirectness, imprecision, and potential publication bias.

Because the included studies were predominantly observational in design, evidence was initially considered low certainty and was further downgraded when substantial heterogeneity, methodological limitations, or residual confounding were identified.

Detailed GRADE assessments for each outcome are provided in Supplementary Table S2.

### 2.8. Data Synthesis

Owing to substantial methodological and clinical heterogeneity among the included studies, a quantitative meta-analysis was not performed. Although several studies investigated similar outcomes, particularly preterm birth and low birth weight, numerical effect estimates suitable for statistical pooling (e.g., odds ratios, risk ratios, hazard ratios, or corresponding confidence intervals) were not consistently reported across studies.

Furthermore, considerable heterogeneity was observed in oral sampling sites, microbiological detection methods, sequencing technologies, microbial targets, study designs, gestational age at sampling, and outcome definitions. Given the absence of consistently reported effect measures and the substantial methodological variability among studies, quantitative synthesis using a random-effects model was considered inappropriate and potentially misleading. Therefore, a qualitative synthesis of the available evidence was performed.

## 3. Results

### 3.1. Study Selection

The literature search identified 1248 records across the selected electronic databases (PubMed, Scopus, Web of Science, and Cochrane Library). After the removal of 314 duplicate records, 934 studies remained for title and abstract screening. Following the initial screening, 822 records were excluded because they did not meet the predefined eligibility criteria.

A total of 112 full-text articles were assessed for eligibility. Among these, 100 studies were excluded, mainly due to the absence of microbiome data, lack of reported pregnancy outcomes, or laboratory-based study designs not involving pregnant women. Ultimately, 10 studies were included in the qualitative synthesis.

### 3.2. Characteristics of Included Studies

The 10 included studies were published between January 2013 and September 2025 and were conducted in Asia and Europe. The included studies comprised cohort, case–control, cross-sectional, longitudinal observational, and nested case–control designs. Some studies have also incorporated bioinformatic functional prediction tools, such as PICRUSt2, to infer the microbial metabolic pathways associated with oral microbial communities. These approaches provide additional insights into the potential functional role of oral dysbiosis in inflammatory processes associated with adverse pregnancy outcomes.

The sample sizes ranged from 15 to 279 participants. Additional study characteristics, including sampling timing, oral sampling sites, and sequencing platforms, are summarized in Supplementary Table S4. Among the included studies, seven investigated associations with preterm birth, five evaluated low birth weight outcomes, and three examined placental microbial colonization or microbial translocation pathways.

The principal periodontal pathogens identified across the studies included *Porphyromonas gingivalis*, *Fusobacterium nucleatum*, and *Prevotella intermedia*. The main characteristics of the included studies, including study design, sample size, microbiological methods, evaluated outcomes, and key findings, are summarized in Table 1.

Detailed study characteristics, including gestational age at sampling, sampling site, and sequencing platform, are provided in Supplementary Table S4.

### 3.3. Maternal Oral Dysbiosis During Pregnancy

Several studies have reported significant alterations in the oral microbial composition during pregnancy [8,10]. An increased abundance of anaerobic periodontal pathogens has been consistently observed during the second and third trimesters, particularly among women with periodontal inflammation [10,12,18,19].

The microorganisms most frequently associated with adverse pregnancy outcomes include *Porphyromonas gingivalis*, *Fusobacterium nucleatum*, and *Prevotella intermedia* [4,6,9].

An increased abundance of *Fusobacterium nucleatum* has been reported in studies investigating preterm birth and placental microbial colonization [8,18,20].

Conversely, reductions in commensal bacterial genera, such as *Neisseria*, have been reported in studies evaluating low-birth-weight pregnancies. Ye et al. demonstrated a significantly lower abundance of *Neisseria* species in mothers delivering low-birth-weight infants, suggesting disruption of oral microbial homeostasis [3].

Longitudinal investigations have further demonstrated progressive increases in oral microbial diversity throughout pregnancy, particularly in the second and third trimesters [2]. These microbial shifts are associated with hormonal fluctuations, altered gingival vascular permeability, and immune modulation during pregnancy [2,4].

The main oral microorganisms associated with adverse pregnancy outcomes, their reported microbiological alterations, and their proposed biological roles are summarized in Table 2.

### 3.4. Association Between Oral Microbiome and Preterm Birth

Several studies have reported the detection of oral bacterial DNA in placental or amniotic samples, supporting the hypothesis of hematogenous microbial dissemination; however, the interpretation of these findings remains controversial because placental microbial signals may be influenced by contamination in low-biomass samples [21,22]. Among these, five studies reported a significantly higher abundance of periodontal pathogens in women who experienced preterm delivery than in term controls.

*Fusobacterium nucleatum* is one of the most consistently reported microorganisms and has been identified in both periodontal and placental samples in multiple studies [7,8,14].

Sequencing-based studies have further demonstrated that global microbial dysbiosis, characterized by reduced microbial stability and increased abundance of pathogenic anaerobic bacteria, is associated with an increased risk of preterm birth.

### 3.5. Association with Low Birth Weight

Five studies investigated the relationship between maternal oral dysbiosis and low birth weight [3,10,13,20,22]. Three studies reported an increased prevalence of periodontal pathogens in mothers who delivered low-birth-weight infants [10,15,20]. Reduced abundance of commensal *Neisseria* species was observed in sequencing-based analyses conducted by Ye et al. [3], whereas increased levels of *Porphyromonas gingivalis* and *Prevotella intermedia* have been associated with inflammatory periodontal conditions in affected pregnancies [10,15]. Collectively, these findings suggest that oral microbial imbalance may be associated with systemic inflammatory pathways that could affect placental function and fetal growth [4,7,14,15].

### 3.6. Risk of Bias Assessment

The methodological quality of the included observational studies was assessed using the Newcastle–Ottawa Scale (NOS) [5].

Overall, most of the included studies reported positive associations between maternal oral dysbiosis and adverse pregnancy outcomes, particularly preterm birth and low birth weight. However, substantial heterogeneity in study design, microbiological assessment methods, and outcome definitions has contributed to variability in the available evidence [9,19,21].

A summary of the NOS scores is presented in Table 3, and detailed domain-level assessments and justifications for individual ratings are provided in Supplementary Table S3.

### 3.7. Certainty of Evidence Assessment

The certainty of evidence was evaluated using the GRADE framework (Supplementary Table S2). Overall, the certainty of evidence was judged to be low for the association between maternal oral dysbiosis and both preterm birth and low birth weight. The certainty of evidence regarding placental microbial colonization and microbial translocation pathways was judged to be very low owing to methodological heterogeneity, limited sample sizes, and ongoing controversy regarding interpretation of microbial signals in low-biomass placental samples.

## 4. Discussion

This systematic review evaluated the current clinical evidence regarding the association between maternal oral dysbiosis and adverse pregnancy outcomes [3,6,7]. These findings suggest that oral dysbiosis during pregnancy may be associated with the systemic inflammatory processes observed in pregnancies complicated by preterm birth and low birth weight [5,7].

Pregnancy is characterized by complex hormonal and immunological changes that influence oral microbial ecology [2]. Increased estrogen and progesterone levels alter gingival vascular permeability and immune responses, facilitating periodontal pathogen proliferation. These microbial shifts may be associated with chronic inflammatory responses in the periodontal tissues [2,6].

One of the most consistent observations across the reviewed studies was the increased prevalence of periodontal pathogens, such as *Porphyromonas gingivalis* and *Fusobacterium nucleatum* [7,9,13,14]. These bacteria produce endotoxins and inflammatory mediators that can enter the systemic circulation. Once disseminated, these microbial products may interact with placental tissues and have been hypothesized to participate in inflammatory pathways associated with premature labor [1,2,7].

Evidence supporting microbial dissemination has emerged from studies detecting oral bacterial DNA in placental and amniotic fluid samples [7,8]. This finding supports the hypothesis that oral pathogens may reach the fetoplacental unit through hematogenous dissemination.

An important consideration when interpreting studies reporting microbial DNA in placental tissues is the ongoing debate regarding the existence of a true placental microbiome. Although several investigations have identified bacterial DNA signatures in placental and amniotic samples, other studies have suggested that these findings may reflect contamination introduced during sample collection, DNA extraction, sequencing procedures, or the analysis of low-biomass specimens [21,22]. Consequently, the detection of bacterial DNA does not necessarily indicate the presence of viable microorganisms or a resident placental microbial community [21,22]. Given these methodological challenges, evidence supporting placental colonization by oral microorganisms should be interpreted with caution. Future studies employing rigorous contamination controls and standardized protocols are required to clarify the biological significance of these placental microbial signals.

However, despite the biological plausibility of these mechanisms, the causal relationship between oral dysbiosis and adverse pregnancy outcomes remains unclear. Many studies included in this review were observational, which limits the ability to establish causality [13,20].

Although most studies achieved low-risk classifications according to the NOS criteria, several investigations were limited by relatively small sample sizes, observational designs, and incomplete adjustment for potential confounding variables. Therefore, risk-of-bias assessments should be interpreted alongside broader methodological limitations, including heterogeneity in microbiological assessment methods and variability in the study populations.

Importantly, the observational nature of the available evidence precludes definitive conclusions about causality. Therefore, the associations identified in this review should not be interpreted as evidence that maternal oral dysbiosis directly causes adverse pregnancy outcomes.

An important challenge in interpreting the relationship between maternal oral dysbiosis and adverse pregnancy outcomes is the potential influence of shared risk factors and residual confounding factors. Several variables, including maternal smoking, obesity, diabetes, socioeconomic status, educational attainment, nutritional status, and access to healthcare, have been associated with periodontal disease and adverse obstetric outcomes. These factors may independently influence oral microbial composition, systemic inflammatory responses, and pregnancy outcomes, potentially contributing to the associations observed in previous studies. Furthermore, adjustment for these confounding variables was not performed consistently across the included studies, limiting the ability to determine whether maternal oral dysbiosis is an independent risk factor or a marker of broader maternal health and social determinants [14,15].

Another important consideration is the potential influence of maternal systemic health and host-related susceptibility factors on periodontal disease and pregnancy outcomes. Metabolic disorders, chronic inflammatory conditions, autoimmune diseases, and genetic susceptibility to periodontal inflammation may influence microbial community structure and inflammatory responses during pregnancy. These factors may act independently or interact with oral dysbiosis, further complicating the interpretation of the observed associations.

Another important consideration is the limited geographic diversity of the available evidence in this review. Most studies included in this review were conducted in China and a small number of European countries, potentially limiting the generalizability of the findings to other populations. The oral microbiome composition may be influenced by dietary habits, cultural practices, oral hygiene behaviors, healthcare access, socioeconomic conditions, environmental exposures, and genetic background. Consequently, associations observed in one population may not be directly applicable to other geographical regions. Future studies should include more diverse populations from underrepresented regions to improve the external validity of the findings regarding maternal oral dysbiosis and pregnancy outcomes.

Future investigations should include broader clinical and laboratory characterizations of pregnant women, including metabolic disorders, autoimmune diseases, inflammatory biomarkers, nutritional status, and genetic susceptibility factors associated with periodontal inflammation. Improved control of comorbidities and systemic inflammatory conditions may strengthen the association between maternal oral dysbiosis and adverse pregnancy outcomes.

Additionally, the genetic and immunological mechanisms contributing to periodontitis development may independently influence the inflammatory pathways involved in pregnancy complications.

Another important limitation identified in the literature is the heterogeneity of microbiome assessment methods. Studies have used different sequencing platforms, microbial sampling techniques, and analytical approaches, which complicates the comparison between studies [14,15].

Advances in bioinformatics and microbiome analysis tools have improved the ability to characterize microbial communities and predict their functions in the human body. For example, computational tools such as PICRUSt2 allow researchers to infer microbial metabolic pathways based on sequencing data, providing insights into the potential mechanisms linking microbial dysbiosis and inflammatory processes [16,17].

Beyond pregnancy outcomes, maternal microbial communities may influence early life microbial colonization in infants. Evidence suggests that maternal oral bacteria may be transmitted vertically to infants, shaping early oral microbial colonization and potentially influencing their long-term health [17,23].

The GRADE assessment indicated that the overall certainty of evidence remains low for the association between maternal oral dysbiosis and adverse pregnancy outcomes. Although several studies have reported consistent associations, the observational nature of the available evidence, methodological heterogeneity, and potential residual confounding reduce confidence in the magnitude and direction of these associations.

From a clinical perspective, these findings highlight the importance of integrating oral health assessments into prenatal care. Preventive dental care and management of periodontal disease during pregnancy may help reduce the systemic inflammatory burden and improve maternal and neonatal health outcomes [1,4].

Another important limitation is the inability to perform a quantitative meta-analysis. Although several studies have evaluated preterm birth and low birth weight, numerical effect estimates have not been consistently reported, and substantial heterogeneity exists in microbiological assessment methods, outcome definitions, and study designs. These factors precluded a meaningful statistical pooling. Consequently, the findings should be interpreted as a qualitative synthesis of the available evidence rather than a quantitative estimate of the effect size.

Future research should prioritize large prospective cohort studies and standardized microbiome analysis methods. Such studies would improve our understanding of the temporal dynamics of oral microbial communities during pregnancy and clarify their roles in adverse pregnancy outcomes.

## 5. Conclusions

This systematic review consolidates the current evidence regarding the association between maternal oral dysbiosis and adverse pregnancy outcomes. These findings suggest that maternal oral dysbiosis may be associated with preterm birth and low birth weight; however, the available evidence remains predominantly observational and does not establish causality. Methodological heterogeneity, variability in microbiological assessment methods, and low overall certainty of evidence limit definitive conclusions. Nevertheless, the findings highlight the potential importance of maternal oral health as a modifiable factor during pregnancy and support the integration of oral health assessments into prenatal care. Future large-scale prospective studies using standardized microbiome methodologies are required to clarify the biological and clinical significance of these associations.

## 6. Limitations

Several limitations should be considered when interpreting the findings of this systematic review. First, most of the included studies were observational in design, including cohort, case–control, and cross-sectional studies. Consequently, the available evidence primarily demonstrates associations rather than causal relationships between maternal oral dysbiosis and adverse pregnancy outcomes.

Second, substantial heterogeneity was observed across studies with respect to oral sampling sites, microbiological assessment methods, sequencing technologies, bioinformatic approaches, gestational age at sampling, and outcome definitions of interest. This variability limits direct comparisons between studies and may have contributed to inconsistencies in the reported findings.

Third, residual confounding remains an important consideration in this study. Several factors, including maternal smoking, obesity, diabetes, socioeconomic status, nutritional status, oral hygiene practices, and access to healthcare, may influence both the oral microbial composition and pregnancy outcomes. Adjustment for these variables was not performed consistently across studies, limiting the ability to determine whether maternal oral dysbiosis is an independent risk factor or a marker of broader maternal health conditions.

Another limitation relates to the geographic distribution of available evidence. Most studies were conducted in China and a limited number of European countries, which may restrict the generalizability of the findings to other populations with different genetic, environmental, cultural, and healthcare characteristics.

Inter-reviewer agreement statistics, such as Cohen’s kappa coefficient, were not prospectively recorded during the screening process. Although independent screening and consensus-based resolution of disagreements were performed, reviewer agreement could not be quantitatively assessed in this study.

The certainty of evidence, evaluated using the GRADE framework, was judged to be low or very low for principal outcomes. This reflects the predominance of observational study designs, methodological heterogeneity, imprecision, and potential influence of residual confounding.

Finally, although several studies have investigated similar pregnancy outcomes, a formal meta-analysis was not feasible because of heterogeneity in study design, microbiological assessment methods, outcome definitions, and inconsistent reporting of effect measures. Consequently, the findings should be interpreted as a qualitative synthesis of the available evidence rather than a quantitative estimate of effect size.

## Figures and Tables

**Table 1:** Characteristics of the Included Studies Evaluating Maternal Oral Microbiota and Pregnancy Outcomes.

Author (Year)	Country	Study Design	Sample Size	Microbiological Method	Main Outcome Evaluated	Key Findings
Ye et al., 2021 [2]	China	Case–control	186 pregnant women	16S rRNA sequencing	Low birth weight	Lower abundance of *Neisseria* spp. associated with low-birth-weight pregnancies
La et al., 2022 [8]	China	Cross-sectional	156 placental samples	Placental microbiome sequencing	Adverse pregnancy outcomes	Placental microbiota associated with pregnancy complications
Collado et al., 2016 [9]	Spain	Observational	15 mother–infant pairs	Placental/amniotic microbiome sequencing	Placental microbial colonization	Distinct microbial communities identified in placenta and amniotic fluid
Ye et al., 2020 [10]	China	Case–control	90 pregnant women	PCR and microbial culture	Preterm birth and low birth weight	Periodontal-related bacteria associated with periodontal inflammation and preterm birth/low-birth-weight pregnancies
Liu et al., 2024 [12]	China	Cohort	111 pregnant women	Oral microbiome sequencing	Low birth weight	Oral microbiome composition predictive of low-birth-weight delivery
La et al., 2022 [13]	China	Longitudinal observational	101 pregnant women	16S rRNA sequencing	Changes in oral microbiome during pregnancy	Oral microbiome composition changed across pregnancy trimesters
Li X et al., 2025 [14]	China	Nested case–control	279 pregnant women	Oral microbiota sequencing	Adverse neonatal outcomes	Maternal oral microbiota associated with adverse newborn outcomes
Šimic et al., 2023 [15]	Croatia	Cohort	152 pregnant women	16S rRNA sequencing	Preterm birth	Increased abundance of *Veillonella*, *Prevotella*, and *Capnocytophaga* in women with preterm birth
Gonzales-Marin et al., 2013 [16]	Spain	Observational	24 mother–infant pairs	ITS sequencing	Preterm birth	*Fusobacterium nucleatum* strains identified in maternal oral and neonatal samples
Pozo et al., 2016 [17]	Spain	Case–control	53 pregnant women	Placental immunohistochemistry and periodontal assessment	Preterm birth and low birth weight	Periodontal disease associated with increased placental inflammatory marker expression in adverse pregnancy outcomes

**Table 2:** Maternal Oral Dysbiosis Associated with Adverse Pregnancy Outcomes.

Microorganism	Reported Alteration	Associated Pregnancy Outcome	Proposed Biological Role	Representative Studies
*Porphyromonas gingivalis*	Increased abundance in periodontal sites during pregnancy	preterm birth, low birth weight	Induction of inflammatory cytokines and periodontal inflammation	Ye et al., 2020 [10]; Liu et al., 2024 [12]
*Fusobacterium nucleatum*	Detection in placental and periodontal samples	preterm birth and placental inflammation	Hematogenous dissemination and placental colonization	La et al., 2022 [8]; Collado et al., 2016 [9]
*Prevotella intermedia*	Increased abundance during pregnancy-associated gingival inflammation	preterm birth, low birth weight	Promotion of inflammatory responses in periodontal tissues	Ye et al., 2020 [10]
*Neisseria* spp.	Reduced abundance in oral microbiota	low birth weight	Loss of commensal microbial balance and oral homeostasis	Ye et al., 2021 [2]
**Placental inflammatory markers associated with periodontal disease**	Increased placental inflammatory response	Preterm birth/low birth weight	Promotion of placental inflammation and adverse fetal outcomes	Pozo et al., 2016 [17]

**Table 3:** Risk of Bias Assessment of Included Studies.

Study	Selection	Comparability	Outcome/Exposure	Total Score	Risk Level
Ye et al., 2021 [2]	4	2	3	9	Low
La et al., 2022 (placental microbiota) [8]	3	1	2	6	Moderate
Collado et al., 2016 [9]	3	1	2	6	Moderate
Ye et al., 2020 [10]	4	2	3	9	Low
Liu et al., 2024 [12]	4	2	3	9	Low
La et al., 2022 (pregnancy longitudinal study) [13]	4	2	2	8	Low
Li X et al., 2025 [14]	4	2	3	9	Low
Šimic et al., 2023 [15]	4	2	3	9	Low
Gonzales-Marin et al., 2013 [16]	3	1	2	6	Moderate
Pozo et al., 2016 [17]	4	2	3	9	Low

Studies scoring 7–9 points were considered low risk of bias, whereas studies scoring 5–6 points were classified as moderate risk of bias.

## Data Availability

No new data were created or analyzed in this study.

## Supplementary Materials

The following supporting information can be downloaded at: https://www.mdpi.com/article/10.3390/jcm15114379/s1, File S1: PRISMA Checklist; File S2: Supplementary Table S1: Complete search strategies used in the systematic review; Supplementary Table S2: GRADE assessment of the evidence; Supplementary Table S3: Detailed Newcastle–Ottawa Scale (NOS) assessment; Supplementary Table S4: Detailed characteristics of included studies.
