# Supplementary material for: Maternal Oral Microbiome Dysbiosis and Adverse Pregnancy Outcomes: Microbial Signatures, Inflammatory Pathways, and Clinical Evidence
Source: J Clin Med. 2026 Jun 5;15(11):4379. doi: 10.3390/jcm15114379 (PMC13257679; doi:10.3390/jcm15114379)
Supplement: Supplementary file 1 [file jcm-15-04379-s001.zip › Supplementary File S2.pdf]

Supplementary Table S1. Complete Search Strategies Used in the Systematic Review

Database	Search Strategy
PubMed/MEDLINE	("Pregnancy"[Mesh] OR pregnancy OR pregnant women) AND ("Oral Microbiome" OR oral microbiome OR oral microbiota OR oral dysbiosis OR periodontal pathogens OR periodontal disease) AND ("Preterm Birth"[Mesh] OR preterm birth OR low birth weight OR adverse pregnancy outcomes OR preeclampsia))
Scopus	TITLE-ABS-KEY (pregnancy OR "pregnant women") AND ("oral microbiome" OR "oral microbiota" OR "oral dysbiosis" OR "periodontal pathogens" OR "periodontal disease") AND ("preterm birth" OR "low birth weight" OR "adverse pregnancy outcomes" OR preeclampsia))
Web of Science	TS= (pregnancy OR "pregnant women") AND ("oral microbiome" OR "oral microbiota" OR "oral dysbiosis" OR "periodontal pathogens" OR "periodontal disease") AND ("preterm birth" OR "low birth weight" OR "adverse pregnancy outcomes" OR preeclampsia)
Cochrane Library	(pregnancy OR pregnant women) AND (oral microbiome OR oral microbiota OR oral dysbiosis OR periodontal pathogens OR periodontal disease) AND (preterm birth OR low birth weight OR adverse pregnancy outcomes OR preeclampsia)

Search period: January 2013 to September 2025.

Final search date: 15 November 2025.

Supplementary Table S2. GRADE Assessment of the Evidence

Outcome	Studies	Study Design	Risk of Bias	Inconsistency	Indirectness	Imprecision	Other Considerations	Certainty
**Preterm birth**	7	Observational	Not serious	Serious	Not serious	Serious	Residual confounding likely	LOW
**Low birth weight**	5	Observational	Not serious	Serious	Not serious	Serious	Residual confounding likely	LOW
**Placental microbial colonization**	3	Observational	Serious	Serious	Serious	Serious	Placental microbiome controversy	VERY LOW

Supplementary Table S3. Detailed Newcastle–Ottawa Scale (NOS) Assessment

Study	Selection (Score)	Selection Justification	Comparability (Score)	Comparability Justification	Outcome/Exposure (Score)	Outcome/Exposure Justification
Ye et al., 2021	4	Representative study population and clearly defined outcomes	2	Consideration of major confounding variables	3	Appropriate microbiological and outcome assessment
Ye et al., 2020	4	Well-defined study groups and participant selection	2	Adjustment for important confounders	3	Reliable exposure and outcome assessment
Liu et al., 2024	4	Representative cohort and adequate follow-up	2	Controlled for major confounding factors	3	Validated outcome assessment methods
La et al., 2022 (placental microbiota)	3	Limited representativeness and observational design	1	Limited adjustment for confounders	2	Acceptable outcome assessment
La et al., 2022 (longitudinal study)	4	Well-defined population and longitudinal follow-up	2	Consideration of major confounders	2	Adequate microbiological assessment
Li X et al., 2025	4	Large sample and appropriate participant selection	2	Adjustment for key confounding variables	3	Robust outcome assessment
Collado et al., 2016	3	Small sample size and observational design	1	Limited control of confounding variables	2	Adequate microbiological assessment
Šimic et al., 2023	4	Representative cohort and clear outcome definitions	2	Adjustment for major confounders	3	Reliable outcome assessment
Gonzales-Marin et al., 2013	3	Small observational cohort	1	Minimal adjustment for confounding	2	Appropriate molecular identification methods
Pozo et al., 2016	4	Clearly defined population and outcomes	2	Consideration of confounding factors	3	Validated histological and clinical assessments

Studies scoring 7–9 points were considered low risk of bias, whereas studies scoring 5–6 points were classified as moderate risk of bias.

Supplementary Table S4. Detailed Characteristics of Included Studies

Abbreviations: NR, not reported or not retrievable from accessible article information; PCR, polymerase chain reaction; qPCR, quantitative polymerase chain reaction; 16S rRNA, 16S ribosomal RNA; ITS, internal transcribed spacer; PLBW, preterm low birth weight; LBW, low birth weight; PTB, preterm birth; TB, term birth; GDM, gestational diabetes mellitus; PROM, premature rupture of membranes.

**Author (Year)**	**Country**	**Study Design**	**Sample Size**	**Gestational Age / Timing of Sampling**	**Sampling Site**	**Microbiological Method**	**Sequencing Platform**	**Outcome Evaluated**	**Key Findings**
Ye et al., 2021	China	Case–control	186 pregnant women	Second trimester (24–28 weeks gestation)	Oral samples; exact site NR	16S rRNA sequencing	Illumina sequencing-based metagenomic and metatranscriptomic analysis	Low birth weight	Lower abundance of Neisseria spp. in the maternal oral microbiome was associated with low-birth-weight pregnancies.
Ye et al., 2020	China	Prospective observational / case–control analysis	90 pregnant women	Second trimester	Saliva; serum for IgG analysis	qPCR for unculturable and culturable periodontal-related bacteria; ELISA for serum IgG	Illumina MiSeq	Preterm low birth weight	Periodontal-related bacteria were associated with periodontal inflammation during pregnancy; Eubacterium saphenum abundance and anti-Aggregatibacter actinomycetemcomitans IgG were associated with PLBW-related indicators.
Liu et al., 2024	China	Cohort	111 pregnant women	Second trimester (24–28 weeks gestation)	Saliva	Oral microbiome sequencing with machine-learning prediction model	Illumina MiSeq	Low birth weight	Oral microbiome composition showed predictive value for low-birth-weight delivery.
La et al., 2022 (placental microbiota)	China	Cross-sectional	156 placental samples	At delivery	Placental tissue	16S rRNA gene sequencing	Illumina MiSeq	Adverse pregnancy outcomes, including GDM and PROM	Placental microbial profiles differed between women with and without selected adverse pregnancy outcomes.
La et al., 2022 (oral microbiota longitudinal study)	China	Prospective longitudinal observational	101 women; 202 unstimulated saliva samples	Preconception and third trimester / late pregnancy	Unstimulated saliva	16S rRNA gene sequencing	Illumina MiSeq	Changes in oral microbiota during pregnancy and oral hygiene associations	Oral microbiota composition changed slightly from preconception to late pregnancy, with more pathogenic taxa in saliva during pregnancy.
Li X et al., 2025	China	Nested case–control	279 pregnant women	Second trimester (24–28 weeks gestation)	Saliva	Oral microbiota sequencing	Illumina MiSeq	Small vulnerable newborns / adverse neonatal outcomes	Maternal oral microbiota was associated with risk of small vulnerable newborn outcomes.
Collado et al.,	Spain	Observational	15 mother–infant	At delivery /	Placenta,	Microbiome	Illumina MiSeq	Placental and	Distinct microbial

2016			pairs	perinatal sampling	amniotic fluid, maternal and neonatal samples	sequencing / bacterial DNA profiling		amniotic microbial colonization	communities were identified in placenta and amniotic fluid; authors proposed that gut colonization may begin prenatally.
Šimic et al., 2023	Croatia	Cohort / case–control comparison	152 pregnant women (61 PTB; 91 TB)	Third trimester (prior to delivery)	Oral swab samples	16S rRNA gene sequencing of the V3–V4 region	Illumina MiSeq	Preterm birth	Oral microbiomes differed between PTB and TB groups; Veillonella, Prevotella, and Capnocytophaga were more abundant in women with preterm birth.
Gonzales-Marin et al., 2013	Spain	Observational	24 mother–infant pairs	At delivery / perinatal sampling	Maternal oral and neonatal samples; exact sites NR	16S–23S rRNA gene intergenic transcribed spacer (ITS) analysis	Illumina MiSeq	Preterm birth / adverse pregnancy outcomes	Fusobacterium nucleatum strains were identified in maternal oral and neonatal samples, supporting possible maternal oral origin in selected adverse outcomes.
Pozo et al., 2016	Spain	Case–control	53 pregnant women	At delivery / placental assessment; oral assessment timing NR	Placental tissue and periodontal clinical assessment	Placental immunohistochemistry and periodontal examination	Placental immunohistochemistry and periodontal examination	Preterm birth and/or low birth weight	Periodontal disease was associated with increased placental inflammatory marker expression in pregnancies complicated by preterm birth and/or low birth weight.

Note: Where gestational age at sampling, exact oral sampling site, or sequencing platform were not clearly available from the accessible article information, the field was marked as NR to avoid unsupported reporting. These entries should be checked against the full-text PDFs before final journal resubmission.
